# Genome-Wide Characterization of Glyceraldehyde-3-Phosphate Dehydrogenase Genes and Their Expression Profile under Drought Stress in *Quercus rubra*

**DOI:** 10.3390/plants13162312

**Published:** 2024-08-20

**Authors:** Hyemin Lim, Michael Immanuel Jesse Denison, Kyungmi Lee, Sathishkumar Natarajan, Tae-Lim Kim, Changyoung Oh

**Affiliations:** 1 Department of Forest Bioresources, National Institute of Forest Science, Suwon 16631, Republic of Korea; kmile@korea.kr (K.L.); ktlmi01@korea.kr (T.-L.K.); happyohcy@korea.kr (C.O.); 2 3BIGS Company Limited, Hwaseong 18469, Republic of Korea; michael@3bigs.com (M.I.J.D.); sathish@3bigs.com (S.N.)

**Keywords:** drought stress, gene expression profile, genome-wide analysis, glyceraldehyde-3-phosphate dehydrogenase, *Quercus rubra*

## Abstract

Glyceraldehyde-3-phosphate dehydrogenase (GAPDH) is crucial in plant metabolism and responses to various abiotic stresses. In the glycolysis pathway, glyceraldehyde-3-phosphate (G3P) is oxidized to 1,3-bisphosphate glycerate (1,3-BPG) through the catalytic action of GAPDH. However, the *GAPDH* gene family in *Quercus rubra* has been minimally researched. In this study, we identified 13 GAPDH-encoding genes in *Q. rubra* through a bioinformatics analysis of genomic data. Evolutionary studies suggest that these *QrGAPDH* genes are closely related to those in *Glycine max* and *Triticum aestivum*. We conducted a comprehensive whole-genome study, which included predictions of subcellular localization, gene structure analysis, protein motif identification, chromosomal placement, and analysis of *cis*-acting regions. We also examined the expression of GAPDH proteins and genes in various tissues of *Q. rubra* and under drought stress. The results indicated diverse expression patterns across different tissues and differential expression under drought conditions. Notably, the expression of Qurub.02G290300.1, Qurub.10G209800.1, and Qrub.M241600.1 significantly increased in the leaf, stem, and root tissues under drought stress. This study provides a systematic analysis of *QrGAPDH* genes, suggesting their pivotal roles in the drought stress response of trees.

## 1. Introduction

Glycolysis is a metabolic pathway that converts glucose into two molecules of pyruvic acid. This process takes place in the cytoplasm, does not require oxygen, and generates ATP energy through substrate-level phosphorylation. Consequently, glycolysis is a crucial pathway that supports metabolic activity under anaerobic conditions. In addition, it supplies ATP, reducing agents, and vital precursors necessary for the growth and development of plants while also facilitating cellular adaptation to cold, drought, and anoxic conditions. [1,2]. Glyceraldehyde-3-phosphate dehydrogenase (EC 1.2.1.12) (GAPDH) is an enzyme that catalyzes the sixth step of glycolysis. In this step, the aldehyde group of glyceraldehyde 3-phosphate is oxidized, and inorganic phosphoric acid is incorporated to form 1,3-bisphosphoglycerate. This reaction enables the reversible transformation of glyceraldehyde 3-phosphate into 1,3-bisphosphoglycerate. Initially, GAPDH was employed as an internal reference for gene expression and protein studies [3]. However, subsequent research has demonstrated that GAPDH is not suitable as an internal reference gene [4,5]. Recent studies have shown that GAPDH is not only involved in glycolysis but also plays a significant role in various abiotic stress responses. For instance, the overexpression of *TaGApC* in *Arabidopsis* has been shown to improve drought tolerance [6]. Similarly, potato and rice plants overexpressing *PsGAPDH* have exhibited increased salt tolerance [7,8]. Furthermore, GAPDH has been found to form immune complexes with protein kinases activated during osmotic stress in salt-treated tobacco cells [9,10]. These findings underscore the pivotal role of GAPDH in enhancing plant tolerance to abiotic stresses [11].

Plants are constantly exposed to abiotic (drought, salt, and carbon dioxide) and biotic stresses from the external environment [12,13,14]. Drought, in particular, is a significant abiotic stressor worldwide that impedes plant growth and production, with profound ecological and economic consequences. Consequently, there is considerable interest in understanding the molecular mechanisms of both drought stress and drought resistance across different plant species. While numerous genes involved in stress signal transduction have been identified, the physiological roles these genes play in stress tolerance and susceptibility remain largely underexplored. The *GAPDH* gene family has been the subject of extensive research across multiple plant species, including *Arabidopsis* [15], rice [8], maize [16], wheat [11], soybean [17], and sweet orange [18]. However, a comprehensive whole-genome analysis has yet to be conducted in northern red oak. The northern red oak, scientifically referred to as *Quercus rubra* L. and commonly recognized by several names, including common red oak, eastern red oak, and mountain red oak, is widely distributed across North America and southeastern Canada [19]. It is a moderate- to fast-growing tree, significant as a lumber source and popular as a shade tree due to its ease of transplant, attractive shape, and dense foliage [19]. The role of GAPDH in regulating the molecular mechanisms of drought stress in *Q. rubra* remains unclear. Therefore, studying the drought resistance mechanisms of red oak and identifying stress-resistant genes is crucial for enhancing the yield and quality of this species. In light of the increasing accessibility of genomic and transcriptomic resources for northern red oak, as provided by the Hardwood Genomics Project [20], this study conducted a thorough analysis of the GAPDH family in red oak.

This study identified 13 members of the GAPDH gene family in *Quercus rubra* and examined their phylogenetic relationships, gene structures, chromosomal positions, and responses to drought stress in leaf, stem, and root tissues. Additionally, we examined the gene expression patterns in leaf, stem, and root tissues of northern red oak and the expression profiles of the *GAPDH* genes under conditions of drought stress and subsequent re-watering. These results provide foundational data for further research on the *QrGAPDH* genes.

## 2. Results

### 2.1. Identification of the Proteins Coded by the GAPDH Gene Family and Physicochemical Characterization of QrGAPDH

A total of 13 GAPDH proteins, each featuring conserved Gp_dh_C and Gp_dh_N domains, have been identified as part of the *Q. rubra* GAPDH family. These proteins have been labeled as GAPDH1-13 according to their chromosomal positions, as outlined in Table 1. The physical and chemical properties of these GAPDH proteins were examined and are summarized in Table 1. The QrGAPDH proteins exhibited variability in length, molecular weight, theoretical isoelectric point, and other characteristics. It is anticipated that the *QrGAPDH* genes will encode polypeptides ranging from 279 to 452 amino acids, with molecular weights spanning from 30.22 to 48.24 kDa. The theoretical pI values varied from 7.02 to 9.46, the aliphatic index ranged from 81.85 to 96.77, and the grand average of hydropathicity for all QrGAPDH proteins was negative, falling between −0.15 and −0.001, which suggests hydrophilic properties. The predicted count of negatively charged residues (Asp + Glu) in the QrGAPDH proteins was between 29 and 46, while the number of positively charged residues (Arg + Lys) ranged from 33 to 53.

**Figure 1:**
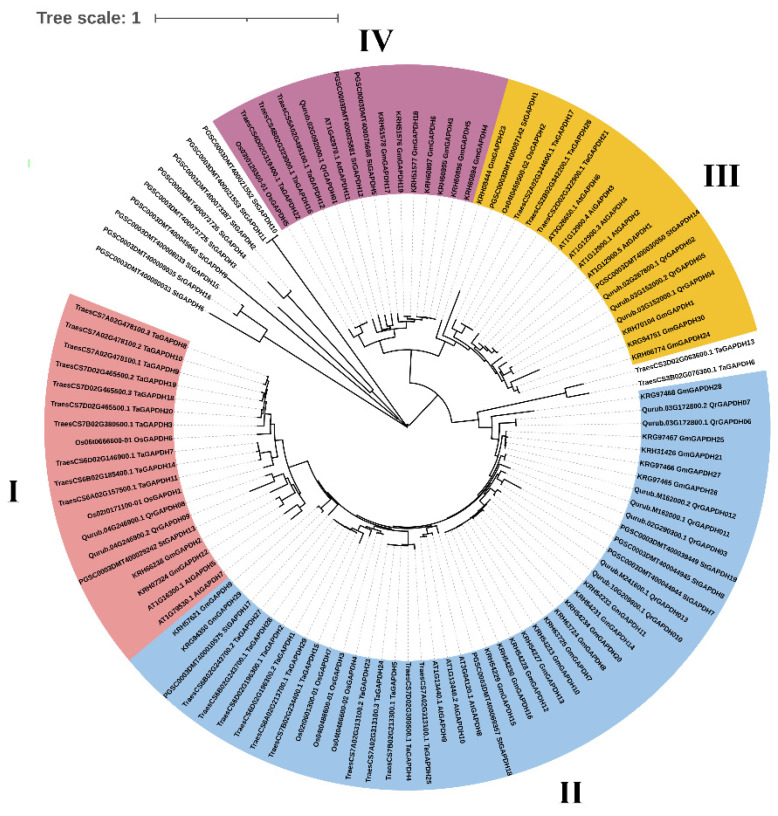
The phylogenetic tree of GAPDH proteins from *A. thaliana*, *O. sativa*, *T. aestivum*, *G. max*, *S. tuberosum* and *Q. rubra*. Red, blue, yellow, and pink color represent the clusters I, II, III, and IV members respectively.

Thirteen *QrGAPDH* genes were discovered within the genome of the red oak. These genes are clearly categorized into four clades (Figure 1), which correspond to four varieties of plant GAPDHs: GAPB, GAPA-2, GAPC2, and GAPCP-2. GAPB comprises a single gene, QrGAPDH01, and is classified within clade IV. GAPA-2 includes three genes—QrGAPDH02, QrGAPDH04, and QrGAPDH05—and falls under clade III. GAPC2, which contains the most genes, includes QrGAPDH03, QrGAPDH06, QrGAPDH07, QrGAPDH10, QrGAPDH11, QrGAPDH12, and QrGAPDH13, and is categorized in clade II. Additionally, GAPCP-2 consists of QrGAPDH08 and QrGAPDH09 and is assigned to clade I (Table 1).

### 2.2. Phylogenetic Tree and Subcellular Localization of GAPDH Genes

In order to clarify the evolutionary connections among GAPDH proteins, a neighbor-joining phylogenetic tree was constructed. This analysis included 13 QrGAPDH proteins in conjunction with those from various species, including *Arabidopsis thaliana*, *Oryza sativa*, *Triticum aestivum*, *Glycine max*, and *Solanum tuberosum*. The resulting phylogenetic tree is illustrated in Figure 1. In this tree, GAPDHs from *A. thaliana* are labeled AtGAPDH1 to AtGAPDH11 (11 genes), those from *O. sativa* as OsGAPDH1 to OsGAPDH7 (7 genes), those from *T. aestivum* as TaGAPDH1 to TaGAPDH29 (29 genes), and those from *S. tuberosum* as StGAPDH1 to StGAPDH19 (19 genes). The GAPDH family proteins from various plants were identified and extracted using an HMM-based approach with the Pfam domains Pfam00044 and Pfam02800, focusing only on complete genes for the comparative analysis. Proteins QrGAPDH08 and QrGAPDH09 were found in clade I, which contained a high number of GAPDHs from *T. aestivum*. Most of the QrGAPDH proteins, specifically QrGAPDH03, QrGAPDH06, QrGAPDH07, QrGAPDH10, QrGAPDH11, QrGAPDH12, and QrGAPDH13, were clustered in clade II along with GAPDHs from other species. QrGAPDH02, QrGAPDH04, and QrGAPDH05 were grouped in clade III with most of the AtGAPDHs, while only QrGAPDH01 was clustered in clade IV (Figure 1).

**Figure 2:**
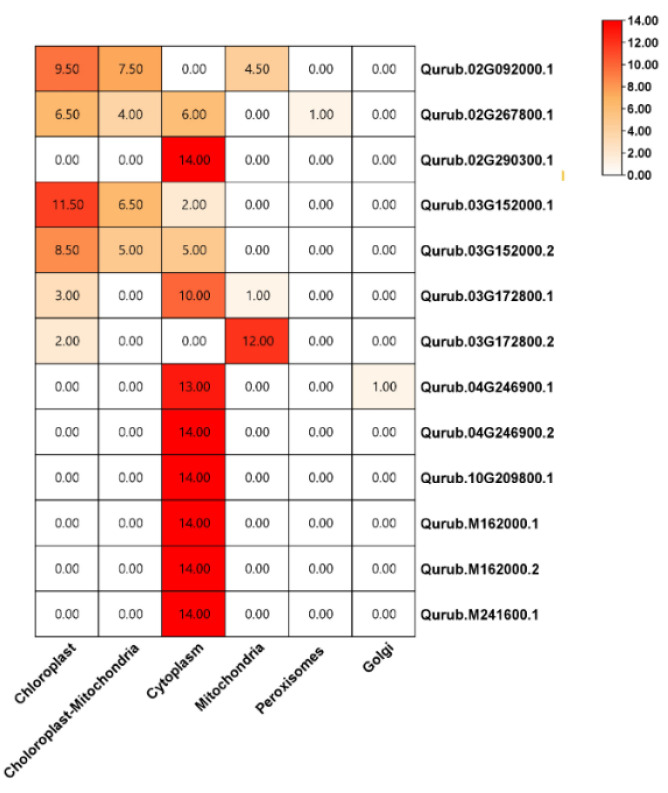
The subcellular distribution of QrGAPDH proteins.

The protein families Qurub.02G092000.1, Qurub.02G267800.1, Qurub.03G152000.1, Qurub.03G152000.2, and Qurub.03G172800.1 were localized in the chloroplast and chloroplast + mitochondria, whereas other proteins such as Qurub.02G290300.1, Qurub.04G246900.1, Qurub.04G246900.2, Qurub.10G209800.1, Qurub.M162000.1, Qurub.M162000.2, and Qurub.M241600.1 were found in the cytoplasm. Additionally, the GAPDH family protein Qurub.03G172800.2 is localized in the mitochondria (Figure 2 and Supplementary Table S1).

### 2.3. Chromosomal Location, Gene Duplication, Collinearity, and Synteny Analysis

**Figure 3:**
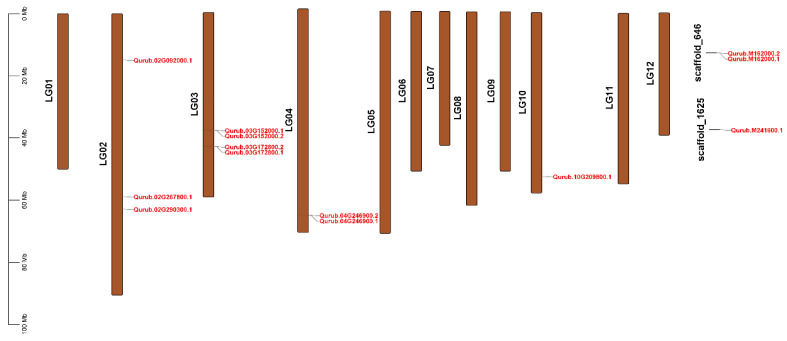
Distribution of GAPDH-encoding genes on chromosomes in *Q. rubra*. The left scale represents the dimensions of each chromosome.

The distribution of *QrGAPDH* genes was not uniform across the 12 chromosomes of *Q. rubra*, as depicted in Figure 3, based on the annotation information from the *Q. rubra* genome. Additionally, two scaffolds, 646 and 1625, also contained a few *QrGAPDH* genes. Ten of the thirteen genes were found on chromosomes 3 (LG03), LG02, LG04, and LG10, while the remaining three genes were located on scaffolds 646 and 1625. Figure 3 illustrates the chromosomal distribution of the 13 *QrGAPDHs* in *Q. rubra*. Details on the chromosome mapping, including gene locations and orientations, are provided in Supplementary Table S2.

In order to investigate the genomic expansion mechanism of the *GAPDH* gene family in *Q. rubra*, we conducted an analysis of the syntenic relationships among the *QrGAPDH* genes. Among the 13 identified *QrGAPDHs*, four genes, which form two distinct gene pairs, displayed syntenic relationships and were found to have undergone tandem duplications (Table 2). The ratio of the substitution rates, denoted as Ka/Ks, serves as a valuable metric for assessing selective pressure during the process of gene duplication. The Ka/Ks ratios for the two identified pairs of tandem duplications, both of which are below 1, indicate that these genes may have experienced purifying selection. It is generally understood that the Ks value is not influenced by natural selection, while the Ka value is subject to its effects. The divergence time for the gene pairs Qurub.02G267800.1 and Qurub.03G152000.2 is estimated to be 95.09 million years ago (Mya), whereas the divergence time for Qurub.02G290300.1 and Qurub.10G209800.1 is approximately 70.80 Mya. The earliest gene duplication event is believed to have occurred 95.09 Mya (Table 2).

**Figure 4:**
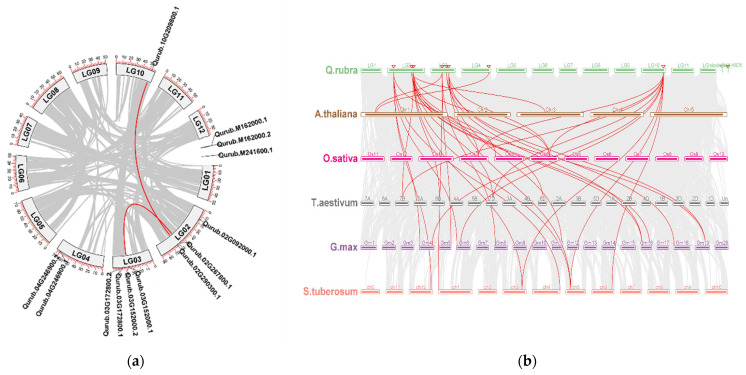
Collinearity and synteny analysis of *QrGAPDH* genes. (**a**) Synteny analysis of *QrGAPDH* genes in *Q. rubra*. Chromosomes are represented as LG along with numbering. Red curves indicated syntenic gene pairs. Gray lines indicate syntenic blocks (**b**) Collinearity analysis of GAPDH genes among *A. thaliana, O. sativa, T. aestivum, G. max* and *S. tuberosum* genomes. Gray lines indicate collinear blocks within the six genomes, while red lines represent collinear GAPDH gene pairs. Red triangles indicate the location of the *GAPDH* genes in *Q. rubra*.

In order to gain a deeper insight into the genetic divergence, gene duplication, and evolutionary patterns of the *GAPDH* gene families across *Q. rubra*, *Arabidopsis*, rice, wheat, potato, and soybean, we examined their syntenic relationships. This examination aimed to identify orthologous *GAPDH* genes among these species utilizing MCScanX. Our analysis revealed the presence of 3, 4, 6, 11, and 8 pairs of orthologous *GAPDH* genes across five comparative assessments: *Q. rubra* vs. *A. thaliana*, *Q. rubra* vs. *O. sativa*, *Q. rubra* vs. *T. aestivum*, *Q. rubra* vs. *G. max*, and *Q. rubra* vs. *S. tuberosum*, respectively (Figure 4).

### 2.4. Gene Structure and Conserved Motifs Analysis of QrGAPDH Genes

**Figure 5:**
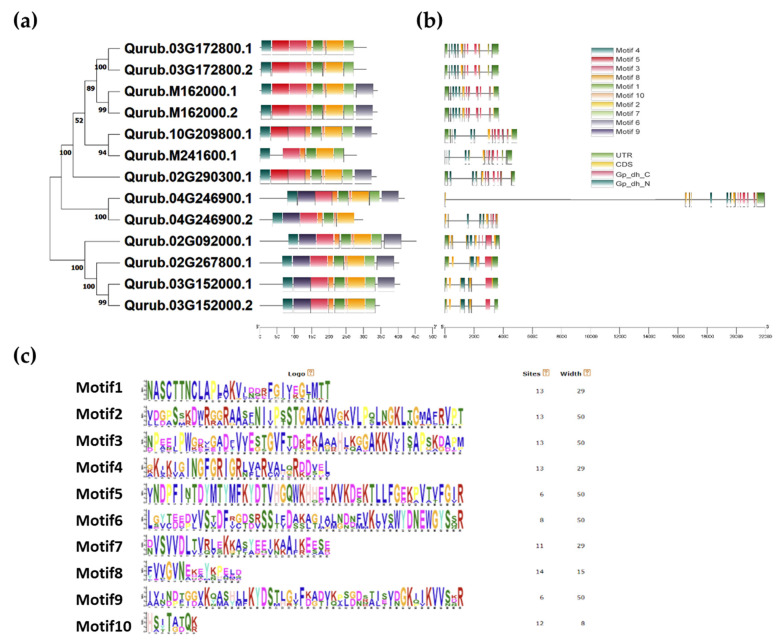
Phylogenetic tree based on the distribution of conserved motifs and exon-intron structure of 13 *QrGAPDHs*. (**a**) Gene evolutionary tree and motif location distribution of *QrGAPDH* genes. (**b**) The gene structure of *QrGAPDH* genes. (**c**) Alignment of repeated sequences in each motif.

In order to understand the sequence characteristics of QrGAPDH proteins in *Q. rubra*, we performed a motif analysis utilizing MEME, which revealed a total of 10 conserved motifs (Figure 5). Proteins belonging to the same phylogenetic group displayed comparable motif compositions and arrangements. For example, the proteins Qurub.03G172800.1, Qurub.03G172800.2, Qurub.M162000.1, Qurub.M162000.2, Qurub.10G209800.1, Qurub.M241600.1, and Qurub.02G290300.1 all contained motif 5. Other proteins, such as Qurub.04G246900.1, Qurub.04G246900.2, Qurub.02G092000.1, Qurub.02G267800.1, Qurub.03G152000.1, and Qurub.03G152000.2, contained motif 9. Motifs 1, 2, 3, 4, and 8 were conserved across all QrGAPDH proteins. Motif 6, which was present only in Qurub.M162000.1, Qurub.M162000.2, Qurub.10G209800.1, Qurub.02G290300.1, Qurub.04G246900.1, Qurub.02G092000.1, Qurub.02G267800.1, and Qurub.03G152000.1, was able to distinguish between variants such as Qurub.04G246900.1/Qurub.04G246900.2 and Qurub.03G152000.1/Qurub.03G152000.2. Motif 7 was also able to discriminate between the Qurub.04G246900.1/Qurub.04G246900.2 variants. Motif 10 was absent in Qurub.M241600.1.

### 2.5. GO Annotation and Cis-Element Analyses of GAPDH Genes in Q. rubra

In order to elucidate the specific roles of GAPDH proteins, a Gene Ontology (GO) annotation was conducted for QrGAPDH proteins. The analysis revealed that the 13 proteins were categorized into three distinct GO categories: molecular function, biological process, and cellular component. Additionally, two distinct groups of GAPC2 proteins were identified. Group I, consisting of Qurub.03G172800.1 and Qurub.03G172800.2, was annotated for the biological process of glucose metabolic process. Regarding their molecular function, these proteins participated in oxidoreductase activity, specifically targeting the aldehyde or oxo groups of donors while utilizing NAD or NADP as acceptors, and were also engaged in the binding of NADP and NAD. Group II, which included Qurub.M162000.1, Qurub.M162000.2, Qurub.10G209800.1, Qurub.M241600.1, and Qurub.02G290300.1, was involved in the glycolytic process as a biological process, localized in the cytoplasm, and exhibited glyceraldehyde-3-phosphate dehydrogenase (NAD^+^) (phosphorylating) activity, with NADP and NAD binding in terms of molecular function. GAPCP-2 protein variants, Qurub.04G246900.1 and Qurub.04G246900.2, were involved in the glucose metabolic process and NAD biosynthetic process as biological processes. They also exhibited nicotinate-nucleotide diphosphorylase (carboxylating) activity and oxidoreductase activity, acting on the aldehyde or oxo group of donors with NAD or NADP as acceptors and in NADP and NAD binding in terms of molecular function. Both GAPA-2 proteins (Qurub.02G267800.1, Qurub.03G152000.1, Qurub.03G152000.2) and the GAPB protein (Qurub.02G092000.1) were involved in the glucose metabolic process as a biological process. They also demonstrated glyceraldehyde-3-phosphate dehydrogenase (NAD^+^) (phosphorylating) activity, indicating the binding of NADP and NAD in relation to their molecular function (Supplementary Table S3).

**Figure 6:**
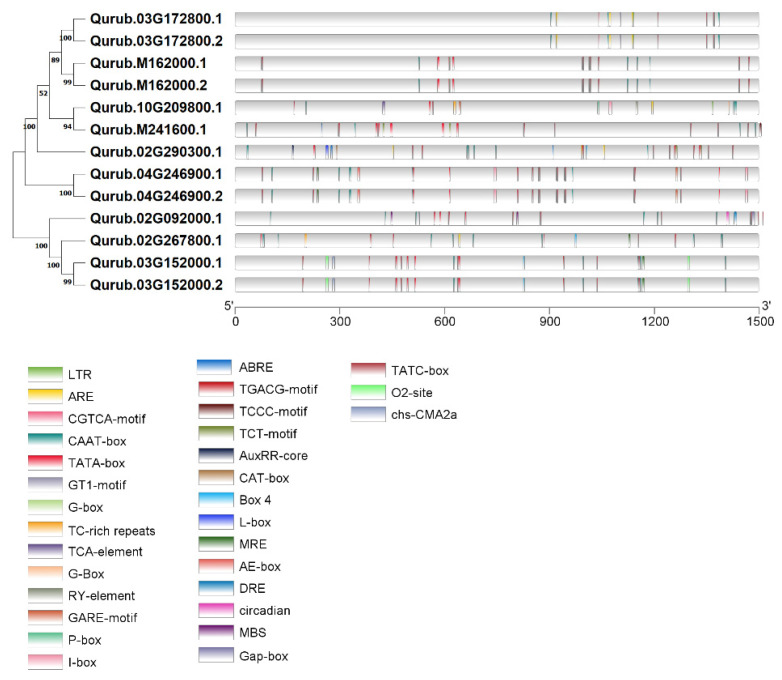
*Cis*-elements in *QrGAPDH* gene promoters.

In order to enhance our understanding of gene regulation mechanisms, we identified *cis*-acting elements within each gene examined (Figure 6). These elements are instrumental in investigating various responses to environmental stress and tissue-specific functions. Utilizing the online resource PlantCARE, we predicted the promoter region located 1500 bp upstream of the *QrGAPDH* genes. Our analysis revealed 23 distinct categories of *cis*-acting elements across 13 *QrGAPDH* genes, which included elements associated with plant hormones such as abscisic acid, salicylic acid, gibberellin, MeJA, and auxin response elements. Specifically, abscisic acid response elements (ABRE) were identified in the genes Qurub.02G290300.1, Qurub.10G209800.1, and Qurub.M241600.1. The salicylic acid response includes the TCA-element found in Qurub.10G209800.1. The gibberellin response element, TATC-box, was located in Qurub.02G267800.1, while the auxin responsive element, AuxRRcore, was present in Qurub.02G290300.1. MeJA response elements, including the CGTCA-motif, were detected in Qurub.03G172800.1, Qurub.03G172800.2, Qurub.M162000.1, and Qurub.M162000.2, and the TGACG-motif was identified in Qurub.03G152000.1, Qurub.03G152000.2, and Qurub.M241600.1. In relation to factors associated with abiotic stress responses, we have identified components linked to defense mechanisms and stress reactions, including those pertaining to dehydration, low temperatures, salt stress, temperature variations, and the MYB binding site that plays a role in drought inducibility. TC-rich repeats, associated with defense and stress responses, were found in Qurub.02G267800.1 and Qurub.10G209800.1. Drought-related elements such as dehydration, low temperature, salt stresses (DRE), and the MYB-binding site (MBS) involved in drought inducibility were identified in Qurub.02G092000.1 (Supplementary Table S4).

### 2.6. Three-Dimensional Structure Modeling and Protein–Protein Interaction Network Analysis

**Figure 7:**
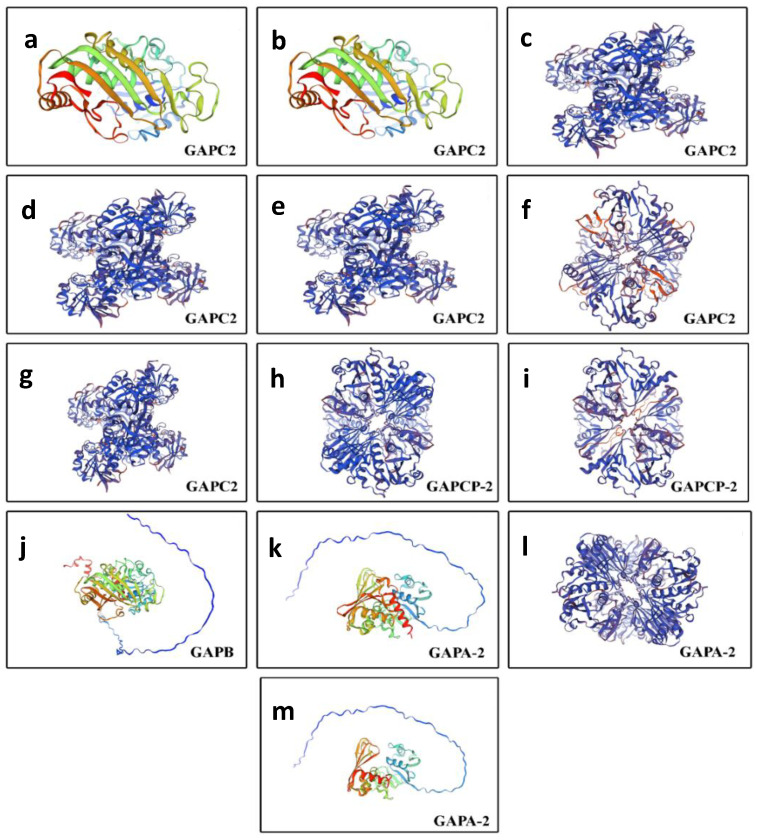
The three-dimensional structure of QrGAPDH proteins was determined through homology modeling via SWISS-MODEL, with the resulting topologies illustrated as cartoons using PyMOL. Each model is accompanied by a quality assessment based on the QMEANDisCo global score. Colors denote the model confidence factor of the SWISS-MODEL server; dark blue regions denote high confidence, green to red gradually lower reliability. The models include (**a**) Qurub.03G172800.1; (**b**) Qurub.03G172800.2; (**c**) Qurub.M162000.1; (**d**) Qurub.M162000.2; (**e**) Qurub.10G209800.1; (**f**) Qurub.M241600.1; (**g**) Qurub.02G290300.1; (**h**) Qurub.04G246900.1; (**i**) Qurub.04G246900.2; (**j**) Qurub.02G092000.1; (**k**) Qurub.02G267800.1; (**l**) Qurub.03G152000.1; (**m**) Qurub.03G152000.2.

To further investigate the newly identified features in QrGAPDH proteins, the three-dimensional (3D) structures of some of these proteins were modeled using the SWISS-MODEL in silico tool. Seven GAPC2 proteins (Qurub.03G172800.1; Qurub.03G172800.2; Qurub.M162000.1; Qurub.M162000.2; Qurub.10G209800.1; Qurub.M241600.1; Qurub.02G290300.1) were identified, and their predicted 3D structures suggest variations in structural topology. For example, the protein variants Qurub.03G172800.1 and Qurub.03G172800.2 exhibit similar topology. Similarly, Qurub.M162000.1, Qurub.M162000.2, Qurub.10G209800.1, and Qurub.02G290300.1 share comparable topological structures, while Qurub.M241600.1 displays a distinct topological structure (Figure 7).

**Figure 8:**
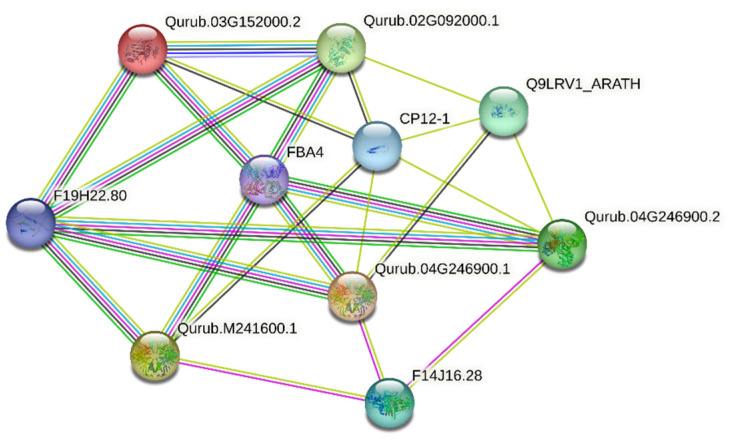
Protein interaction network of QrGAPDH. The color scales represent the relative signal intensity scores.

The two GAPCP-2 protein variants, Qurub.04G246900.1 and Qurub.04G246900.2, shared similar topological structures. Among the three GAPA-2 proteins—Qurub.02G267800.1, Qurub.03G152000.1, and Qurub.03G152000.2—Qurub.03G152000.1 exhibited a distinct topology. Among the 13 QrGAPDH proteins, only five were involved in protein–protein interactions: Qurub.02G092000.1, Qurub.02G092000.1 (repeated, possibly an error), Qurub.03G152000.2, Qurub.04G246900.1, Qurub.04G246900.2, and Qurub.M241600.1 (Figure 8). To achieve a deeper the understanding of the protein–protein interaction network results, five distinct clusters were identified. In Cluster I, the protein Qurub.02G092000.1 was found to interact with Qurub.03G152000.2, FBA4, Q9LRV1_ARATH, CP12-1, and F19H22.80. Qurub.02G092000.1 and Qurub.03G152000.2 were closely connected, with a confidence score of 0.983.

In cluster II, Qurub.03G152000.2 interacted with proteins including FBA4, F19H22.80, and CP12-1. In clusters III, IV, and V, the protein variants Qurub.04G246900.1 and Qurub.04G246900.2 interact with the same members, such as FBA4, CP12-1, F14J16.28, F19H22.80, and Q9LRV1_ARATH (Table 3).

### 2.7. Expression Profiles of QrGAPDH Genes under Drought Stress and Re-Watering in Various Tissues

**Figure 9:**
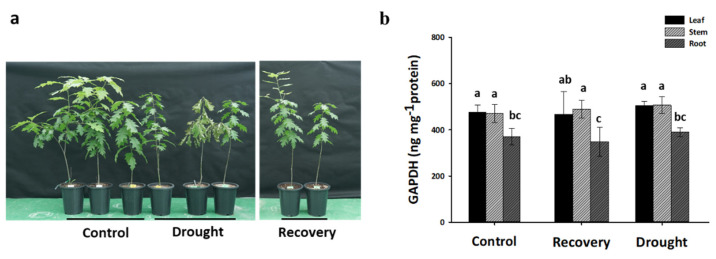
The growth phenotype and expression of the GAPDH protein of *Q. rubra*. (**a**) Phenotype of a red oak seedling for 1 month of drought stress and recovery (6 days after re-watering). (**b**) GAPDH protein content under drought stress in different tissues. The outcomes of the tests conducted in triplicate are presented as means ± standard deviation (SD). Distinct lowercase letters denote significant differences (*p* < 0.05).

In order to investigate the function of GAPDH in the response of *Q. rubra* to drought conditions, we analyzed the GAPDH protein levels in the leaf, stem, and root tissues while also assessing the growth phenotypes. It was confirmed that the oak seedlings exhibited phenotypic differences under drought stress conditions; however, they recovered upon re-watering. The GAPDH protein was consistently expressed in all tissues, with significantly lower levels observed in the roots compared to other tissues. Additionally, there was no variation in protein content in response to either drought stress or re-watering (Figure 9).

**Figure 10:**
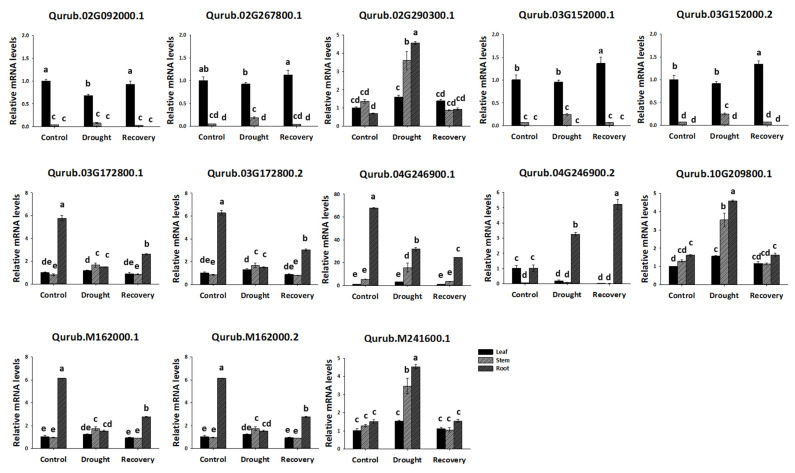
Relative expression profiles of 13 *QrGAPDH* genes under drought stress and in different tissues. The standard deviation determined from three biological replicates is shown in error bars. Significant differences (*p* < 0.05) are indicated by different lowercase letters.

qPCR analysis was conducted to further investigate the expression of *QrGAPDH* across various tissues. All 13 *QrGAPDH* genes showed expression in different tissues, aligning with the results from the protein assay (Figure 10). The expression of *QrGAPDH* genes varied among the leaf, stem, and root tissues. Specifically, Qurub.02G092000.1, Qurub.02G267800.1, Qurub.03G152000.1, and Qurub.03G152000.2 were prominently expressed in the leaves and localized in the chloroplasts (Figure 2). Notably, Qurub.03G172800.1, Qurub.03G172800.2, Qurub.04G246900.1, Qurub.M162000.1, and Qurub.M162000.2 showed high expression levels in the roots (Figure 10). Tissue-specific genes can be utilized to specifically express a desired tissue by utilizing the promoter of the gene. These genes could be utilized to explore tissue-specific expression patterns, including leaf-specific and root-specific expressions. The genes associated with root-specific expression may play an essential role in the glycolytic pathway of *Q. rubra*. Additionally, Qurub.02G290300.1, Qurub.10G209800.1, and Qurub.M241600.1 demonstrated high expression in all examined tissues (Figure 10), suggesting their potential use as internal controls.

After 30 days of drought treatment, the relative expression levels of Qurub.02G290300.1, Qurub.10G209800.1, and Qurub.M241600.1 were significantly increased compared to the control. In contrast, the expression levels of Qurub.03G172800.1, Qurub.03G172800.2, Qurub.04G246900.1, Qurub.M162000.1, and Qurub.M162000.2 were markedly reduced compared to the control, especially in the root tissue. Interestingly, the relative expression levels of Qurub.02G092000.1, Qurub.02G267800.1, Qurub.03G152000.1, and Qurub.03G152000.2 showed no difference under drought stress (Figure 10). These results establish a theoretical foundation for additional research into the molecular properties and biological roles of QrGAPDH.

## 3. Discussion

GAPDH is a crucial enzyme involved in the glycolysis pathway. Studies indicate that GAPDH significantly contributes to plant development and their reactions to environmental stressors, especially salinity and drought [8]. Genome-wide analyses of the *GAPDH* gene family have been performed across several plant species, such as *Arabidopsis* [21], soybean [17], wheat [11], sweet orange [18], watermelon [22], and basil [23]. Nevertheless, the molecular function of GAPDH in red oak (*Q. rubra* L.) remains unreported. The selection of proteins from the GAPDH family was based on the identification of two essential domains: the Gp_dh_N domain (PF00044) and the Gp_dh_C domain (PF02800). Only 13 members possessing both complete domains were selected, while those with incomplete or truncated domains were excluded. We conducted a comprehensive analysis of these 13 *GAPDH* gene members in *Q. rubra* using the latest genome assembly. This analysis included their phylogenetic relationships, gene structure, conserved motifs, chromosomal positions, molecular 3D structure predictions, and expression profiles under drought stress. The physicochemical properties of the primary protein sequences are instrumental in determining their structural and biological functions. All but one of the QrGAPDH proteins were found to be basic; the exception (Qurub.02G290300.1) was neutral. These proteins had an average amino acid composition of 351 and a molecular weight of 37,880 kDa. The average hydropathy value, determined using GRAVY, was negative, indicating that the proteins were hydrophilic. The aliphatic index confirmed the thermostability of the proteins identified in this study. The average instability index of the GAPDH proteins, at 25.48, indicates their stability (values greater than 40 indicate stability; values less than 40 indicate instability). Overall, the structural and functional characteristics of QrGAPDH corresponded with those found in other species.

The phylogenetic examination of GAPDH family members in Q. rubra, along with other documented species including *A. thaliana*, *O. sativa*, *T. aestivum*, *G. max*, and *S. tuberosum*, indicates that the genes encoding GAPDH in these species have a shared ancestral lineage. The constructed phylogenetic tree reveals that *QrGAPDH* shares a close evolutionary relationship with *G. max* and *T. aestivum*. Four clusters were observed, with clade II (blue) being the largest, clades I (red) and III (yellow) being equal in size, and clade IV (pink) being the smallest. Genes located within the same clade exhibit similar expression levels. For instance, the genes clustered within the blue shades, Qurub.02G290300.1, Qurub.10G209800.1, and Qurub.M241600.1, display similar expression patterns, whereas, the pairs Qurub.03G172800.1/Qurub.03G172800.2 and Qurub.M162000.1/Qurub.M241600.1 in the blue cluster, show similar expression patterns. In the yellow cluster (clade III), genes such as Qurub.02G267800.1, Qurub.03G152000.1, and Qurub.03G152000.2 exhibit similar gene expression. The pink cluster (clade IV) contains only one *Q. rubra* gene. However, in the red cluster (clade I), genes such as Qurub.04G246900.1 and Qurub.04G246900.1 did not show similar expression patterns among multiple genes from the same clade, suggesting that the GAPDH genes have undergone duplication, resulting in functional redundancy. According to a previous report, GAPDH proteins are localized in both the cytoplasm and plastid [24]. The members of the *GAPDH* gene family in *Arabidopsis thaliana*, including AtGAPA-1, AtGAPA-2, and AtGAPB, are recognized for their localization in chloroplasts and their role in the photosynthetic reductive carbon cycle. Similarly, the QrGAPDH members within the same blue clade, comprising QrGAPDH08 and QrGAPDH09, are also predicted to perform analogous functions [25]. Furthermore, additional family members, including AtGAPC-1 and AtGAPC-2, have been recognized as cytosolic glycolytic enzymes within the same clade as QrGAPDH03, QrGAPDH06, QrGAPDH10, QrGAPDH11, QrGAPDH12, and QrGAPDH13. However, the localization of proteins at the subcellular level can provide insights into their functional roles within the cell. We report that a total of eight GAPDH proteins were localized in the cytoplasm, three in the chloroplast, one in both the chloroplast and cytoplasm, and another in the mitochondria. Reports indicate that proteins associated with glycolysis and gluconeogenesis metabolism exhibited a notable accumulation in response to drought stress. The swift and substantial activation of GAPDH is likely to facilitate ongoing energy production and provide signals for the associated metabolic processes [26]. GAPDH maintains its essential function as a glycolytic “housekeeping” protein within the cytoplasm; however, growing evidence suggests that posttranslational modifications of cytosolic GAPDH direct this protein towards functional pathways that diverge from glycolysis [27]. The expression of the *GAPDH* gene in aspen was found to be elevated following drought stress. Similarly, in wheat, the expression of TaGAPDH12 in shoots showed a significant increase after the plants were subjected to four different abiotic stresses, namely cold, heat, salt, and drought [11]. Whole-Genome Duplication (WGD) significantly contributed to the evolution of the *QrGAPDH* gene family, in conjunction with segmental duplication. Additionally, analyses of the nonsynonymous and synonymous substitution ratios (Ka and Ks) of orthologous *GAPDH* gene pairs were conducted to identify the underlying mechanisms driving the evolution of the *GAPDH* gene family. The findings indicated that the majority of orthologous *GAPDH* gene pairs exhibited a Ka/Ks ratio of less than 1, which implies the presence of purifying selection throughout the evolution of the *GAPDH* gene family and the preservation of these genes’ functions. Collinearity analysis identified two segmental duplications, revealing that *QrGAPDH* failed to maintain a conserved duplication. However, segmental duplication was advantageous in extending the *GAPDH* genes in *Q. rubra*.

We assessed the *cis*-acting elements in relation to their involvement in gene function and regulatory mechanisms [28]. Plant promoters serve as crucial regulatory elements necessary for the transcription of plant genes and hold significant regulatory functions at the transcriptional level. In our analysis of the 1500 bp putative promoter regions of all *GAPDHs*, we identified a variety of *cis*-regulatory elements. The majority of *GAPDHs* exhibited different types of *cis*-elements associated with plant hormones, such as abscisic acid response elements, salicylic acid response elements, gibberellin response elements, MeJA response elements, and auxin response elements. Furthermore, various *cis*-regulatory elements that play a role in defense mechanisms, stress responses, circadian rhythms, and MYB binding sites are associated with the regulation of genes responsible for flavonoid biosynthesis. Flavonoids have the potential to enhance drought tolerance in maize seedlings by mitigating oxidative damage caused by drought conditions and by modulating stomatal movements [29]. The DRE and ABRE elements are recognized for their involvement in the regulation of protein and hormone activities in reaction to cold and drought stress [30]. It has been proposed that MYB proteins interact with critical MREs to elevate the expression of genes related to phenylpropanoid biosynthesis and stress response [31]. The genes Qurub.02G092000.1 and Qurub.03G152000.2 are associated with the *cis*-regulatory element, MYB binding site, which is involved in drought inducibility (MBS). Three genes showed differential expression in drought-induced trees, including Qurub.M241600.1, Qurub.02G290300.1, and Qurub.10G209800.1. Notably, Qurub.02G290300.1 and Qurub.10G209800.1 contain drought-responsive *cis*-acting elements such as MYB, a transcription factor whose expression is regulated by drought conditions [32,33].

*QrGAPDH* genes exhibited varying expression levels across different tissues under drought stress. The expression pattern of the GAPDH gene was analyzed through protein content and qPCR analyses to determine how red oak GAPDH responds to water deficiency stress. The promoter sequence of QrGAPDH includes *cis*-elements such as ABRE and TC-rich repeats (Figure 6). These findings demonstrate that drought stress significantly induces the expression of QrGAPDH (Figure 10). The findings offer valuable information regarding the possible functions of *QrGAPDH* genes and their expression patterns across different tissues in response to drought conditions. Comprehensive genome-wide analyses, along with gene expression studies under drought stress, may assist plant biologists and breeders in identifying and functionally characterizing the *QrGAPDH* genes that enhance stress tolerance in red oak cultivars.

## 4. Materials and Methods

### 4.1. Identification of the GAPDH Genes in Q. rubra

The genomic sequence and annotation information for *Quercus rubra* were sourced from Phytozome version 13 (https://phytozome-next.jgi.doe.gov/info/Qrubra_v2_1; accessed on 26 June 2023) [34]. These data were used to identify the GAPDH genes in *Q. rubra*. To locate these genes, an HMMER search was conducted in the *Q. rubra* protein database using an HMM profile. This profile comprises an N-terminal NAD(P)-binding domain, identified as the Gp_dh_N domain (PF00044), along with a C-terminal catalytic domain, designated as the Gp_dh_C domain (PF02800). Both domains are derived from the Pfam database (https://www.ebi.ac.uk/interpro; accessed on 26 June 2023). The E-value cut-off was set at 10^−5^. The presence of conserved GAPDH domains was verified through a batch CD search against the Pfam database (https://www.ncbi.nlm.nih.gov/; accessed on 26 June 2023), focusing exclusively on complete proteins. Subsequently, the physical and chemical characteristics of the GAPDH family members were examined utilizing the ExPASy-ProtParam tool (https://web.expasy.org/protparam/; accessed on 26 June 2023) [35].

### 4.2. Phylogenetic Relationships, Gene Structure, and Conserved Motif Analyses of the GAPDH Genes

Domain alignments were executed using MEGA (version X) and subsequently visualized through iToL (https://itol.embl.de/; accessed on 26 June 2023) [36,37]. The multiple sequence alignments of GAPDH proteins were carried out with ClustalW. To construct the phylogenetic tree, the neighbor-joining method was applied, incorporating pairwise deletion and 1000 bootstrap replications. The exon/intron structures of the *GAPDH* genes were analyzed using the Gene Structure Display Server 2.0 (http://gsds.cbi.pku.edu.cn/; accessed on 26 June 2023) alongside genomic sequence and structural annotation data [38]. Additionally, MEME (http://meme-suite.org/; accessed on 26 June 2023) was employed to investigate the conserved motifs of the GAPDH proteins, with the maximum limit for motif recognition set at 10 [39,40].

### 4.3. Chromosomal Distribution, Syntenic Analysis, Homology Modeling, and Predicting the Protein–Protein Interaction Network of the GAPDH Genes

The Generic Feature Format Version 3 (GFF3) file, which provides positional and structural data regarding genes located on the chromosomes of Q. rubra, was acquired from Phytozome v13 (https://phytozome-next.jgi.doe.gov/info/Qrubra_v2_1; accessed 26 June 2023) [34]. Following this, TBTools (http://mg2c.iask.in/mg2c_v2.0/; accessed on 26 June 2023) was employed to map the *GAPDH* genes to their respective chromosomes. The syntenic relationships among the *GAPDH* genes in *A. thaliana*, *O. sativa*, *T. aestivum*, *G. max*, and *S. tuberosum* were examined and illustrated using the Multiple Collinearity Scan Toolkit (MCScanX) and TBtools [41]. Genomic data for *A. thaliana*, *O. sativa*, *T. aestivum*, *G. max*, and *S. tuberosum* were sourced from Ensembl Plants (https://plants.ensembl.org/index.html; accessed on 26 June 2023). The downstream analysis feature in MCScanX was utilized to compute the Ka and Ks values for segmental and tandem duplicate gene pairs. The Ks values were subsequently applied to estimate the timing of duplication events (T) using the formula T = Ks/2λ, where λ = 1.5 × 10^−8^ s for dicots [42]. Additionally, the Ka/Ks ratio was analyzed to ascertain the selection mode of the *GAPDH* genes [43]. The three-dimensional structure of QrGAPDH proteins was modeled through a homology modeling technique in SWISS-MODEL. The functions of AtGAPDHs, which are predicted to be orthologous to QrGAPDHs, were explored using NCBI (https://www.ncbi.nlm.nih.gov/; accessed on 26 June 2023). To further investigate the relationships among the QrGAPDH proteins, the orthologous proteins of QrGAPDHs in Arabidopsis were utilized to predict the protein–protein interaction network. STRING (https://string-db.org/; accessed on 25 September 2023) was used to develop the functional interaction network of these proteins [44].

### 4.4. GO, Nr Annotation, and Analyses of Cis-Regulatory Elements of the QrGAPDHs

The NCBI database was chosen as the primary reference for this research, and the Blast2GO software was employed to perform Gene Ontology (GO) analysis on the *QrGAPDH* genes [45]. Annotations from the Nr database were obtained through the NCBI BLASTP tool, which can be accessed at (https://blast.ncbi.nlm.nih.gov/Blast.cgi?PROGRAM=blastp&PAGE_TYPE=BlastSearch&LINK_LOC=blasthome; accessed on 26 June 2023). To identify the cis-acting elements, the 1500 bp sequence located upstream of the coding regions of the QrGAPDH genes was extracted and analyzed using PlantCARE, which is available at (http://bioinformatics.psb.ugent.be/webtools/plantcare/html/; accessed on 28 June 2023) [46].

### 4.5. Plant Materials and Drought Stress Treatment

The two-year-old *Q. rubra* seedlings were grown in a greenhouse at the National Institute of Forest Science (NIFOS) in Korea, situated at 37°15′04″ N, 136°57′59″ E. These seedlings were placed in topsoil that was irrigated until the soil moisture content reached 40% before the commencement of the drought stress treatment. Following this, the plants experienced a 30-day period without irrigation, which was succeeded by a re-watering phase lasting six days. Control plants were maintained under the same conditions, except that their soil moisture levels were consistently kept at 40%. Soil moisture was monitored bi-daily using a moisture probe from ICT International Pty. Ltd., located in Armidale, NSW, Australia. Upon collection, samples were immediately frozen in liquid nitrogen and stored at −80 °C until further analysis. Each treatment group included three biological replicates.

### 4.6. Measurement of GAPDH Contents

To assess the levels of GAPDH, approximately 0.1 g samples were obtained from different tissues. The GAPDH concentration in two-year-old seedling plants was determined utilizing the Plant GAPDH ELISA Kit (MyBioSource, San Diego, CA, USA) in accordance with the manufacturer’s instructions. Absorbance readings were taken at 450 nm with an automatic plate reader (SpectraMax M2, Molecular Devices, San Jose, CA, USA).

### 4.7. RNA Extraction and Quantitative Real-Time PCR (qPCR)

For transcriptional analysis of GAPDH genes in response to drought stress, total RNAs were isolated from *Q. rubra* using the Beniprep^®^ Super Plant RNA extraction kit (InVirusTech Co., Gwangju, Republic of Korea). RNA samples were then converted into single-stranded cDNA using the cDNA EcoDry Premix (TaKaRa, Shiga, Japan). qPCR was conducted on a CFX96 Touch Real-Time PCR Detection System (Bio-Rad, Hercules, CA, USA) using IQtm SYBR Green Supermix (Bio-Rad, Hercules, CA, USA). The reaction conditions were as follows: an initial denaturation at 95 °C for 30 s, followed by 38 cycles of 95 °C for 5 s and 60 °C for 34 s. The relative abundance of transcripts was analyzed using the 2^−ΔΔCt^ method [47]. To normalize the qPCR results, the expression levels of *α-tub* and *18S rRNA* were used [48,49]. The gene-specific primers employed are detailed in Supplementary Table S5.

### 4.8. Statistical Analysis

A one-way ANOVA was employed for the analyses of qPCR and GAPDH protein levels. To assess multiple comparisons, the Tukey test for honestly significant differences (HSD) was applied. The criterion for statistical significance was set at *p* < 0.05. Results are presented as means ± standard deviations (SD).

## 5. Conclusions

This paper presents a genome-wide analysis of 13 *QrGAPDH* genes in the *Q. rubra* genome. These genes exhibit varied expression patterns across different tissues, with the expression levels of some *QrGAPDH* genes being influenced by drought and subsequent re-watering. Analyzing the genome of *QrGAPDH* genes aids in understanding the crucial factors that regulate gene expression in *Quercus*. The expression of GAPDHs is modulated by various hormones (ABA, ET, SA, and MeJA) and abiotic stressors (oxidative stress, salt, drought, and cold), as evidenced by promoter *cis*-element analysis and qPCR detection. Additionally, Qurub.M241600.1, Qurub.02G290300.1, and Qurub.10G209800.1 demonstrated significant differential expression under drought conditions, suggesting a potential role for *QrGAPDHs* in drought tolerance. In conclusion, our study provides a comprehensive understanding of the properties of *QrGAPDHs* and establishes a foundation for further research into the biological functions of the *Q. rubra* gene family.

## Figures and Tables

**Table 1:** Gene IDs and physicochemical properties of the 13 GAPDH-encoding genes in *Q. rubra*.

Gene ID	Gene Symbol	Database ID	Number of AAs ^z^	MW ^y^ (KDa)	Theoretical pI ^x^	Total Number of Negatively Charged Residues (Asp + Glu)	Total Number of Positively Charged Residues (Arg + Lys)	Instability Index	Aliphatic Index	GRAVY ^w^
QrGAPDH01	GAPB	Qurub.02G092000.1	452	48,239.24	8.82	29	33	27.65	91.44	−0.031
QrGAPDH02	GAPA-2	Qurub.02G267800.1	402	42,859.98	8.60	32	37	24.91	96.77	−0.001
QrGAPDH03	GAPC2	Qurub.02G290300.1	337	36,689.02	7.02	34	41	24.78	90.24	−0.103
QrGAPDH04	GAPA-2	Qurub.03G152000.1	404	43,237.28	8.46	34	42	26.59	95.54	−0.018
QrGAPDH05	GAPA-2	Qurub.03G152000.2	346	36,881.19	9.46	35	43	28.18	95.55	−0.060
QrGAPDH06	GAPC2	Qurub.03G172800.1	308	33,677.08	9.25	39	43	20.55	90.49	−0.106
QrGAPDH07	GAPC2	Qurub.03G172800.2	307	33,606.00	9.25	42	45	20.58	90.46	−0.112
QrGAPDH08	GAPCP-2	Qurub.04G246900.1	418	44,648.66	8.48	42	45	35.19	83.95	−0.101
QrGAPDH09	GAPCP-2	Qurub.04G246900.2	298	31,696.12	8.86	43	45	31.22	81.85	−0.107
QrGAPDH10	GAPC2	Qurub.10G209800.1	338	36,616.96	8.25	43	45	21.75	91.66	−0.072
QrGAPDH11	GAPC2	Qurub.M162000.1	340	37,073.58	8.22	43	47	22.51	90.82	−0.113
QrGAPDH12	GAPC2	Qurub.M162000.2	339	37,002.50	8.22	44	47	22.55	90.8	−0.119
QrGAPDH13	GAPC2	Qurub.M241600.1	279	30,216.82	9.03	46	53	24.85	92.22	−0.147

^z^ AA, amino acid; ^y^ MW, molecular weight; ^x^ pI, isoelectric point; ^w^ GRAVY, grand average of hydropathy.

**Table 2:** The Ka/Ks ratios of duplication for *QrGAPDHs*.

Gene 1	Gene 2	Ka	Ks	Ka_Ks	Divergence Time (Mya ^z^)	Duplication Type
Qurub.02G267800.1	Qurub.03G152000.2	0.076964373	1.304651722	0.058992275	95.09123335	Tandem
Qurub.02G290300.1	Qurub.10G209800.1	0.065133869	0.971411426	0.067050755	70.80258209	Tandem

^z^ Mya, million years ago.

**Table 3:** Interacting proteins and their confidence score.

Cluster	Node 1	Node 1 ID	Node 2	Node 2 ID	Score
Cluster I	Qurub.02G092000.1	3702.P25857	Qurub.03G152000.2	3702.Q9LPW0	0.983
			FBA4	3702.F4KGQ0	0.839
			Q9LRV1_ARATH	3702.Q9LRV1	0.758
			CP12-1	3702.O22914	0.714
			F19H22.80	3702.Q9SVJ5	0.568
Cluster II	Qurub.03G152000.2	3702.Q9LPW0	Qurub.02G092000.1	3702.P25857	0.983
			FBA4	3702.F4KGQ0	0.839
			F19H22.80	3702.Q9SVJ5	0.568
			CP12-1	3702.O22914	0.432
Cluster III	Qurub.04G246900.1	3702.Q9SAJ6	FBA4	3702.F4KGQ0	0.879
			CP12-1	3702.O22914	0.857
			F14J16.28	3702.F4I3I1	0.559
			F19H22.80	3702.Q9SVJ5	0.512
			Q9LRV1_ARATH	3702.Q9LRV1	0.439
Cluster IV	Qurub.04G246900.2	3702.Q5E924	FBA4	3702.F4KGQ0	0.880
			CP12-1	3702.O22914	0.857
			F14J16.28	3702.F4I3I1	0.559
			F19H22.80	3702.Q9SVJ5	0.512
			Q9LRV1_ARATH	3702.Q9LRV1	0.400
Cluster V	Qurub.M241600.1	3702.Q9FX54	FBA4	3702.F4KGQ0	0.873
			CP12-1	3702.O22914	0.870
			F19H22.80	3702.Q9SVJ5	0.512
			F14J16.28	3702.F4I3I1	0.425

## Data Availability

The data presented in this study are available on request from the corresponding author.

## Supplementary Materials

The following supporting information can be downloaded from https://www.mdpi.com/article/10.3390/plants13162312/s1: Table S1: Subcellular localization of QrGAPDHs and the number of proteins present in each organelle. Table S2: Chromosome mapping of *QrGAPDH* genes, their location, and orientation. Table S3: List of QrGAPDHs detected in *Quercus rubra* through the HMMER approach and NR-based GO annotation using BLAST2GO. Table S4: List of *cis*-regulatory elements identified across 13 *QrGAPDH* genes. Table S5: Sequence of primers used for qPCR in *Quercus rubra*.
